# Case report: Recovery from refractory myasthenic crisis to minimal symptom expression after add-on treatment with efgartigimod

**DOI:** 10.3389/fneur.2024.1321058

**Published:** 2024-01-22

**Authors:** Keiko Watanabe, Shinichi Ohashi, Takuya Watanabe, Yuki Kakinuma, Ryuta Kinno

**Affiliations:** ^1^Division of Neurology, Department of Internal Medicine, Showa University Northern Yokohama Hospital, Yokohama, Japan; ^2^Respiratory Disease Center, Showa University Northern Yokohama Hospital, Yokohama, Japan

**Keywords:** myasthenic crisis, efgartigimod, minimal symptom expression, intravenous immunoglobulins, plasmapheresis

## Abstract

Myasthenic crisis, a life-threatening exacerbation of myasthenia gravis, is a significant clinical challenge, particularly when refractory to standard therapies. Here, we described a case of myasthenic crisis in which the patient transitioned from refractory myasthenic crisis to minimal symptom expression after receiving add-on treatment with efgartigimod, a novel neonatal Fc receptor antagonist. A 54 years-old woman who was diagnosed with anti-acetylcholine receptor antibody-positive myasthenia gravis experienced respiratory failure necessitating mechanical ventilation. Despite aggressive treatment with plasmapheresis, intravenous immunoglobulins, and high-dose corticosteroids, her condition continued to deteriorate, culminating in persistent myasthenic crisis. Efgartigimod was administered as salvage therapy. Remarkable improvement in neuromuscular function was observed within days, allowing for successful weaning from mechanical ventilation. Over the subsequent weeks, the patient’s symptoms continued to ameliorate, ultimately reaching a state of minimal symptom expression. Serial assessments of her serum anti-acetylcholine receptor antibody titer showed a consistent decline in parallel with this clinical improvement. This case highlights efgartigimod’s potential as an effective therapeutic option for refractory myasthenic crisis, offering new hope for patients facing this life-threatening condition.

## Introduction

Myasthenic crisis (MC) is the most severe, life-threatening manifestation of myasthenia gravis (MG), often requiring noninvasive and/or mechanical ventilation, supportive enteral feeding, and intensive care unit management (1). Disease-modifying treatments such as plasmapheresis and intravenous immunoglobulins (IVIG) can be administered in the management of MC, but these treatments are not effective in all patients. IVIG and plasmapheresis are more likely to be combined sequentially for the refractory MC (2).

In recent years, the pathogenesis of MG has become clearer, and more targeted therapies are being developed (3). Monoclonal antibodies (mAbs) now offer a very attractive therapeutic approach to MG because they can specifically and effectively target several immunopathological pathways, including the complement cascade, B-cell-associated differentiation group proteins, and human neonatal Fc receptors (FcRn). To date, the C5-directed mAb eculizumab and the FcRn inhibitor efgartigimod have been approved for the chronic treatment of anti-acetylcholine receptor (AChR) antibody-positive MG. However, the efficacy of these agents in MC remains unknown. We present a case of MC in which the patient transitioned from refractory MC to minimal symptom expression (MSE), defined as a MG activities of daily living (MG-ADL) scale of 0 or 1, after she receiving add-on treatment with efgartigimod. This case suggests that efgartigimod may be a viable treatment option for MC.

## Case presentation

At 5 months prior to the present event, the patient (a 54 years-old Japanese woman) had been diagnosed with anti-AChR antibody-positive MG. She had a history of lumbar disc herniation but was not receiving treatment for it. Her initial presenting symptoms were drooping eyelids and double vision. A thymectomy was performed for her non-invasive thymoma (Type B1, Masaoka stage II) 2 months prior to her presentation. Treatment with oral steroid therapy (prednisolone 5 mg/day), tacrolimus (3 mg/day), and pyridostigmine (60 mg 3×/day) resulted in complete resolution of her clinical symptoms [MG-ADL scale: 0; MG composite (MGC) scale: 0]. Serum anti-glutamic acid decarboxylase (GAD) antibody was measured for possible comorbid stiff-person syndrome (SPS) but was negative (<5.0 U/mL).

During her routine clinic follow-up, she noticed weakness in her neck muscles, along with a recurrence of drooping eyelids and double vision. She was admitted to our hospital due to progressive neck-muscle weakness (day 0). On admission, percutaneous oxygen saturation was recorded at 97% (room air); all other vital signs were normal. She exhibited drooping eyelids, double vision, dysarthria, dysphagia, and hypernasality. Manual muscle test scores were 2 for neck flexion and extension, and 3 for shoulder abduction. The MG-ADL and MGC scale results were 17 and 32, respectively. Blood sample testing was positive for anti-AChR antibodies (28.0 nmol/L) and negative for anti-muscle-specific tyrosine kinase (MuSK) antibodies (<0.02 nmol/L). Blood gas analysis showed normal findings (PaO_2_: 74.3 mmHg; PaCO_2_: 39.0 mmHg).

**Figure 1:**
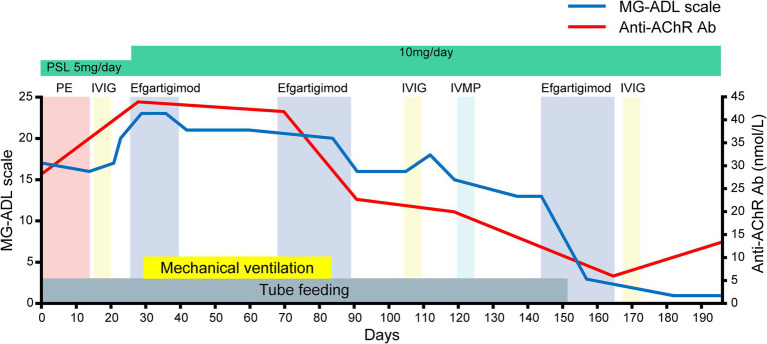
The patient’s clinical course after admission as assessed by the myasthenia gravis-activities of daily living (MG-ADL) scale. The timing of each therapy, the MG-ADL results, and the titer of anti-AChR antibody (anti-AChR Ab) are shown. Day 0: the day of hospital admission. Note that serial assessments of the patient’s serum anti-AChR antibody titers paralleled the clinical improvement assessed by the MG-ADL scale after efgartigimod administration. IVIG, intravenous immunoglobulin; IVMP, intravenous methylprednisolone; PE, plasmapheresis; PSL, prednisolone.

**Figure 2:**
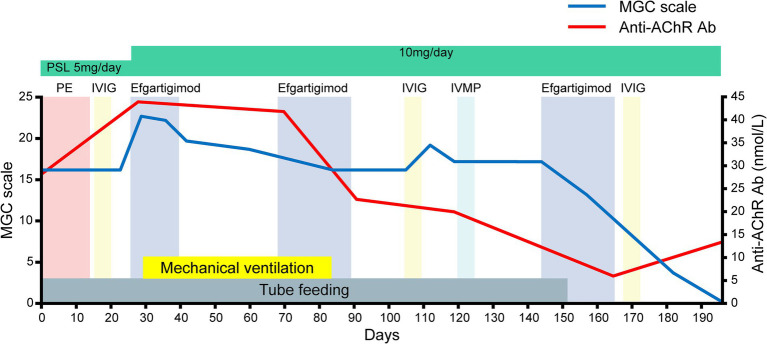
The patient’s clinical course after admission as assessed by the myasthenia gravis composite (MGC) scale. Note that the serial assessments of the patient’s serum anti-AChR antibody titers paralleled the clinical improvements assessed by the MGC scale after efgartigimod administration.

After admission (Figures 1, 2), the patient required tube feeding due to severe dysphagia. Unfortunately, there was no way to administer tacrolimus via a feeding tube in our setting, and the patient’s tacrolimus medication was discontinued. In accord with the recommendation of the Japanese guidelines (4), we decided to perform the first-acting treatments. We initiated plasma exchange therapy. Following the first plasma exchange, the patient experienced anaphylactic shock, leading to the implementation of immunoadsorption plasmapheresis. However, after six plasmapheresis sessions the patient’s clinical symptoms showed no improvement, and limb muscle weakness had developed. On day 15, we also administered IVIG therapy (0.4 g/kg/day × 5 days), but the patient’s clinical symptoms worsened, resulting in respiratory failure (MG-ADL scale: 20; MGC scale: 32).

On day 24, we commenced the first cycle of intravenous efgartigimod (10 mg/kg/week, 4 infusions per cycle) as immunosuppressive therapy. To intensify the immunosuppressive therapy, we suggested increasing the dose of oral prednisone, but the patient refused to increase the dose beyond 10 mg. On day 29 (15 days after the IVIG), her respiratory failure worsened (PaO_2_: 93.0 mmHg; PaCO_2_: 73.6 mmHg), necessitating mechanical ventilation. The first cycle of efgartigimod was limited to three infusions due to ventilator-associated pneumonia. Following the intravenous efgartigimod therapy, the patient’s limb muscle weakness and neck muscle weakness gradually resolved on days 42 and 48, respectively.

On day 66, the second cycle of intravenous efgartigimod was administered. The patient’s dyspnea gradually improved after the second cycle of treatment, and on day 84, she was successfully weaned from mechanical ventilation. She was able to walk approx. 50 meters on her own. On day 105 (39 days after the second cycle of intravenous efgartigimod), she noticed muscle weakness in her neck and upper limbs. Considering the possibility of prolonged effects of the initial IVIG for her initial improvement, we initiated the second IVIG treatment (0.4 g/kg/day × 5 days), which proved ineffective. Intravenous corticosteroids (1 g/day × 5 days), which was standard therapy for MC, were also ineffective. Based on the above data, we considered for the first time that efgartigimod could be effective for improving the clinical symptoms of MC. We thus initiated the third cycle of intravenous efgartigimod on day 143. After completing three cycles of intravenous efgartigimod, the patient’s dysphagia gradually improved, and she was able to tolerate oral intake very well.

**Figure 3:**
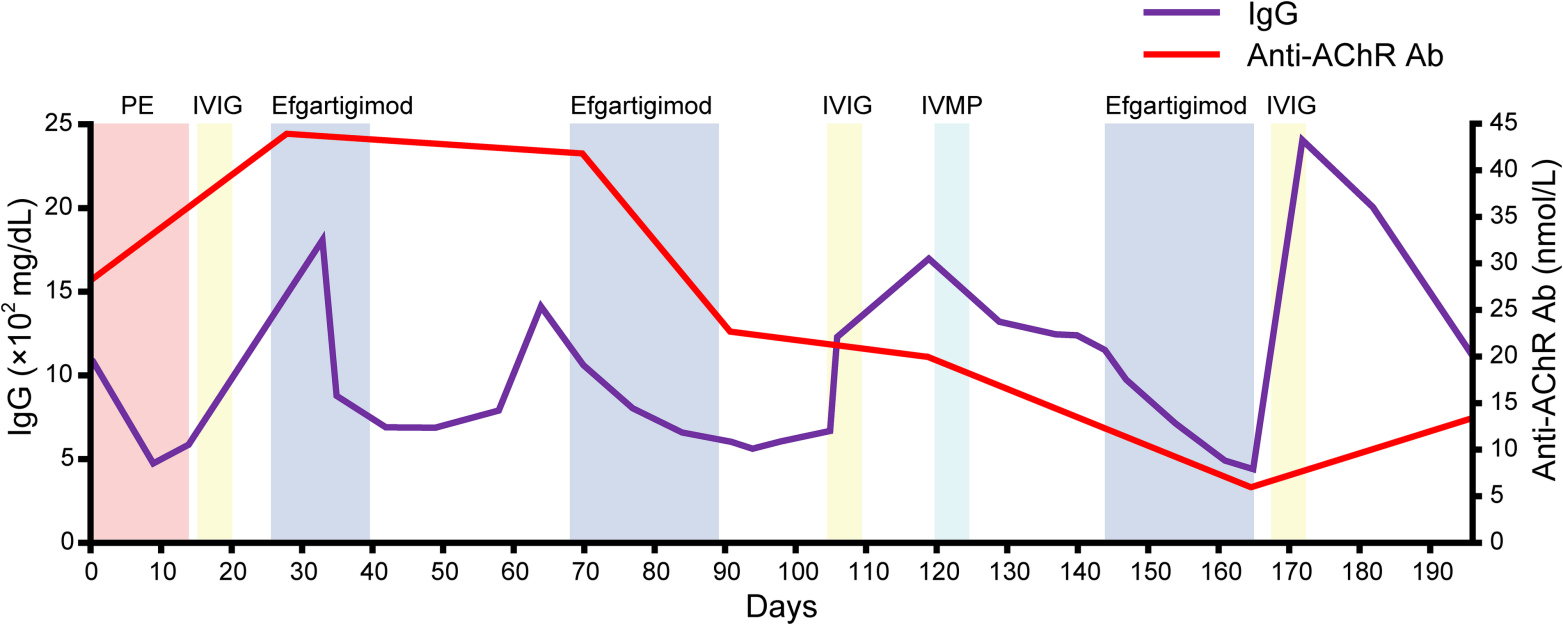
The serial data of the IgG concentration and anti-AChR antibody. Note that the serum IgG concentration was decreased after both plasmapheresis and intravenous efgartigimod, whereas anti-AChR antibodies were decreased after the intravenous efgartigimod only.

She was discharged on day 182. Serial assessments of her serum anti-AChR antibody titers showed a consistent decline in parallel with her clinical improvement. In addition, her serum IgG concentration was decreased after both plasmapheresis and intravenous efgartigimod, whereas anti-AChR antibodies were decreased after intravenous efgartigimod only (Figure 3). Based on this clinical course, we consider this a case of refractory MC that responded well to efgartigimod. At the first outpatient visit after discharge (on day 196), the patient had maintained the MSE status. Follow-up chest computed tomography (~6 months after the tumor removal) showed no evidence of recurrence of the thymoma.

## Discussion

This patient, a 54 years-old woman, presented with severe weakness and promptly underwent the disease-modifying therapies considered standard first-line treatments for MG exacerbation, including plasmapheresis and IVIG (5). However, her clinical symptoms did not resolve following these interventions. A response to treatment is typically observed within 2 days of plasmapheresis and within 4–5 days of IVIG (6). In the case of IVIG, it has been noted that the therapeutic effect may appear over a relatively long period of time — as long as 28 days (7). In our patient’s case, MG exacerbation persisted even after 2 weeks of IVIG, ultimately leading to MC. Moreover, her ventilator management continued until day 85. These clinical observations suggest that (i) the patient had refractory MC, and (ii) standard first-line therapies alone were inadequate for her treatment.

Intravenous efgartigimod is the first FcRn antagonist therapy approved in several countries worldwide for the chronic management of MG (8, 9). FcRn plays a central role in IgG homeostasis by rescuing IgGs from lysosomal degradation. Efgartigimod disrupts this IgG recycling process by binding to the FcRn, thereby reducing the levels of IgG, including anti-AChR antibodies, in the blood. Improvement can typically be observed within the first 1 to 2 weeks after injection. Because of this relatively immediate effect, it is thought that efgartigimod may be effective against MC (1). Improvement in our patient’s MC was noted about 12 days after the first intravenous efgartigimod administration, suggesting the effectiveness of this therapy for MC. This clinical progress aligned with the serial assessments of the patient’s serum anti-AChR antibody titers, further supporting the efficacy of this therapy for her case. Moreover, her status transitioned from MC to MSE after three cycles of intravenous efgartigimod, indicating the potential effectiveness of multi-cycle therapy for the treatment of MC. The interval before the third cycle was slightly longer because it took time to determine whether efgartigimod was effective for improving the symptoms of MC. The reduced time in the cycle may improve the efficacy of the drug.

The patient’s serum anti-AChR levels decreased after the intravenous efgartigimod treatment, in contrast to the standard first-line treatment (Figure 3), and this correlated with the improvement in her clinical symptoms (Figures 1, 2). These findings suggest that the addition of intravenous efgartigimod was effective and rapid in reducing IgG in this patient compared to the standard first-line treatment alone, such as plasmapheresis or IVIG. Regarding the patient’s plasmapheresis, it is surprising that the plasmapheresis was ineffective while efgartigimod was effective, despite their similar mechanisms of action.

Therapeutically, plasmapheresis works by filtering circulating proteins and antibodies, whereas efgartigimod accelerates the catabolism of IgG (10). The anti-AChR antibodies belong to the IgG1–IgG3 class (11), and the depletion of IgG constitutes the primary objective of therapies designed to mitigate the pathophysiological repercussions of IgG autoantibodies binding to their targets. The efficacy of plasmapheresis in successfully depleting pathogenic targets hinges on various factors, including the size and half-life of the target molecule, its distribution within compartments (intravascular vs. extravascular), and the volume and frequency of plasma exchange (12). Due to the diminutive size and protracted half-life of IgG, multiple plasmapheresis procedures are imperative to eliminate IgG from the circulation and the extravascular space, with an estimated requirement of approx. six procedures to achieve a reduction in circulating IgG levels by ~60%–70% (12). Such procedures induce a precipitous decline in circulating IgG autoantibodies, potentially triggering an augmented production of autoantibodies (i.e., an anti-AChR antibody overshoot) following therapeutic plasmapheresis (13), leading to a resurgence of disease activity.

It is established that the increase in antibody levels observed after plasmapheresis likely reflects a reduction in catabolism, coupled with an unchanged rate of synthesis occurring in the extravascular space (e.g., spleen) (14). Moreover, when performing plasmapheresis, an invasive procedure is required for vascular access, and there are non-negligible problems associated with venous access, such as cardiovascular adverse events and infections (15). In contrast, treatment with FcRn inhibitors requires minimally invasive intervention and may be less contingent on the compartmental localization of IgG, effectively targeting both intravascular and extravascular IgG for lysosomal degradation, due to the nearly ubiquitous expression of FcRn (16). In addition, since the plasma concentrations of therapeutic antibodies persist for long periods of time (17), IgG depletion may be more sustained than the short-term effects of plasmapheresis. Another possibility is that only the anti-AChR antibodies in the intravascular space are removed by plasmapheresis, leading to an overshoot in the production of anti-AChR antibodies. In our patient’s case, the intravenous efgartigimod may have affected the extravascular space, resulting in a comprehensive reduction of serum anti-AChR antibody levels, which in turn contributed to the clinical improvements.

IVIG requires a minimally invasive procedure, similar to efgartigimod. However, a problem with IVIG is the lack of volunteer blood donors, since IVIG is a product of plasma processing. IVIG has limited availability and relies on blood donors, which contributes to supply challenges (18). The limited availability is also because IVIG is used for many other indications (chronic inflammatory demyelinating polyneuropathy, hematology, etc.). In this respect, efgartigimod has an advantage over IVIG because it does not require blood from a donor.

A recent study described excellent results regarding MG complicated by SPSs successfully treated with efgartigimod; these patients experienced very large reductions in MG-ADL scores (19). This evidence opens the possibility of treating patients affected by anti-GAD antibody-related diseases, which are also often found in SPS. Since our patient also showed a decrease in the MG-ADL scale (Figure 1) and MGC scale (Figure 2), the coexistence of autoimmune diseases may need to be considered. Since our patient did not have anti-GAD antibodies and did not have the typical clinical manifestations of SPS such as progressive and fluctuating muscle rigidity (20), the possibility that SPS was at least a complication is negative. Nevertheless, clinicians should suspect SPS when a patient improves rapidly with efgartigimod, as in the present case, because of the risk of the underdiagnosis of SPS in patients with MG.

It should be noted that the effect of efgartigimod observed in our patient’s case may be an “add-on” effect to other conventional therapies. Especially considering the duration of the effect onset of IVIG (7), it is difficult to interpret the course of our patient’s case as an effect of efgartigimod alone. In addition, it must be emphasized that the report presented here is a single case study; comprehensive investigations involving larger patient cohorts and experimental studies are needed to elucidate the underlying mechanisms and to develop this potential new therapeutic strategy for managing MC. Nonetheless, the observations from this case have promising implications for clinicians encountering cases of refractory MC.

## Data availability statement

The raw data supporting the conclusions of this article will be made available by the authors, without undue reservation.

## Ethics statement

Ethical approval was not required for the studies involving humans because this is a case report and no experimental procedures were performed. The studies were conducted in accordance with the local legislation and institutional requirements. The participants provided their written informed consent to participate in this study. Written informed consent was obtained from the individual(s) for the publication of any potentially identifiable images or data included in this article.

## Author contributions

KW: Conceptualization, Data curation, Investigation, Writing – original draft, Writing – review & editing. SO: Data curation, Writing – review & editing. TW: Data curation, Writing – review & editing. YK: Data curation, Writing – review & editing. RK: Conceptualization, Data curation, Funding acquisition, Supervision, Validation, Writing – original draft, Writing – review & editing.

## References

[1] Claytor B Cho SM Li Y. Myasthenic crisis. Muscle Nerve. (2023) 68:8–19. doi: 10.1002/mus.27832 37114503

[2] Neumann B Angstwurm K Mergenthaler P Kohler S Schonenberger S Bosel J. Myasthenic crisis demanding mechanical ventilation: a multicenter analysis of 250 cases. Neurology. (2020) 94:e299–313. doi: 10.1212/WNL.0000000000008688 31801833

[3] Vanoli F Mantegazza R. Antibody therapies in autoimmune neuromuscular junction disorders: approach to myasthenic crisis and chronic management. Neurotherapeutics. (2022) 19:897–910. doi: 10.1007/s13311-022-01181-3, PMID: 35165857 PMC9294078

[4] Murai H Utsugisawa K Motomura M Imai T Uzawa A Suzuki S. The Japanese clinical guidelines 2022 for myasthenia gravis and Lambert–Eaton myasthenic syndrome. Clin Exp Neuroimmunol. (2023) 14:19–27. doi: 10.1111/cen3.12739

[5] Gajdos P Chevret S Clair B Tranchant C Chastang C. Clinical trial of plasma exchange and high-dose intravenous immunoglobulin in myasthenia gravis. Myasthenia Gravis Clinical Study Group. Ann Neurol. (1997) 41:789–96. doi: 10.1002/ana.410410615 9189040

[6] Gold R Schneider-Gold C. Current and future standards in treatment of myasthenia gravis. Neurotherapeutics. (2008) 5:535–41. doi: 10.1016/j.nurt.2008.08.011, PMID: 19019304 PMC4514693

[7] Zinman L Bril V. IVIG treatment for myasthenia gravis: effectiveness, limitations, and novel therapeutic strategies. Ann N Y Acad Sci. (2008) 1132:264–70. doi: 10.1196/annals.1405.038 18567877

[8] Howard JF Jr Bril V Vu T Karam C Peric S Margania T. Safety, efficacy, and tolerability of efgartigimod in patients with generalised myasthenia gravis (ADAPT): a multicentre, randomised, placebo-controlled, phase 3 trial. Lancet Neurol. (2021) 20:526–36. doi: 10.1016/S1474-4422(21)00159-9 34146511

[9] Bhandari V Bril V. FcRN receptor antagonists in the management of myasthenia gravis. Front Neurol. (2023) 14:1229112. doi: 10.3389/fneur.2023.1229112, PMID: 37602255 PMC10439012

[10] Mina-Osorio P Tran M-H Habib AA. Therapeutic plasma exchange versus FcRn inhibition in autoimmune disease. Transfus Med Rev. (2023) 38:150767. doi: 10.1016/j.tmrv.2023.150767 37867088

[11] Rødgaard A Nielsen F Djurup R Somnier F Gammeltoft S. Acetylcholine receptor antibody in myasthenia gravis: predominance of IgG subclasses 1 and 3. Clin Exp Immunol. (1987) 67:82–8. PMID: 3621677 PMC1542559

[12] Rossing N. Intra- and extravascular distribution of albumin and immunoglobulin in man. Lymphology. (1978) 11:138–42. 739785

[13] Ching J Richards D Lewis RA Li Y. Myasthenia gravis exacerbation in association with antibody overshoot following plasmapheresis. Muscle Nerve. (2021) 64:483–7. doi: 10.1002/mus.27341 34076268

[14] Charlton B Schindhelm K. The effect of extracorporeal antibody removal on antibody synthesis and catabolism in immunized rabbits. Clin Exp Immunol. (1985) 60:457–64. 3893813 PMC1577210

[15] Ipe TS Davis AR Raval JS. Therapeutic plasma exchange in myasthenia gravis: a systematic literature review and meta-analysis of comparative evidence. Front Neurol. (2021) 12:662856. doi: 10.3389/fneur.2021.662856, PMID: 34531809 PMC8439193

[16] Kim J Hayton WL Robinson JM Anderson CL. Kinetics of FcRn-mediated recycling of IgG and albumin in human: pathophysiology and therapeutic implications using a simplified mechanism-based model. Clin Immunol. (2007) 122:146–55. doi: 10.1016/j.clim.2006.09.001, PMID: 17046328 PMC2791364

[17] Pyzik M Sand KMK Hubbard JJ Andersen JT Sandlie I Blumberg RS. The neonatal Fc receptor (FcRn): a misnomer? Front Immunol. (2019) 10:1540. doi: 10.3389/fimmu.2019.01540, PMID: 31354709 PMC6636548

[18] Pavlekovics M Engh MA Lugosi K Szabo L Hegyi P Terebessy T. Plasma exchange versus intravenous immunoglobulin in worsening myasthenia gravis: a systematic review and meta-analysis with special attention to faster relapse control. Biomedicine. (2023) 11:3180. doi: 10.3390/biomedicines11123180, PMID: 38137401 PMC10740589

[19] Di Stefano V Alonge P Rini N Militello M Lupica A Torrente A. Efgartigimod beyond myasthenia gravis: the role of FcRn-targeting therapies in stiff-person syndrome. J Neurol. (2023) 271:254–62. doi: 10.1007/s00415-023-11970-1, PMID: 37682316 PMC10769952

[20] Toro C Jacobowitz DM Hallett M. Stiff-man syndrome. Semin. Neurol. (1994). 14:54–8. doi: 10.1055/s-2008-1041073 7984830

